# Impact of the COVID-19 Pandemic on Children’s Physical Activity As Perceived by Their Parents

**DOI:** 10.7759/cureus.80703

**Published:** 2025-03-17

**Authors:** Heba W Haidar, Alaa R Kalash, Fatima A Alshamsi, Noura N Alzaabi, Amal Hussein

**Affiliations:** 1 General Practice, Tawam Hospital, Abu Dhabi, ARE; 2 General Practice, Emirates Health Services, Sharjah, ARE; 3 General Practice, Sheikh Shakhbout Medical City, Abu Dhabi, ARE; 4 Family and Community Medicine, University of Sharjah, Sharjah, ARE

**Keywords:** children, covid-19 pandemic, exercise, lockdown effects, parental involvement, physical activity, physical inactivity, play, screen time, sedentary behavior

## Abstract

Background

Since the outbreak and global spread of COVID-19, countries rapidly introduced a range of preventative measures and isolation protocols to ensure safety, which ultimately led to the implementation of total lockdowns. As a result, children lost access to spaces where they typically engage in physical activity and were required to stay indoors.

Objective

The objective of this study is to assess the impact of the COVID-19 pandemic on children’s physical activity levels and analyze the factors influencing their physical activity during this period.

Methods

Parents from various Emirates participated in an online survey to evaluate changes in their children's physical activity during the COVID-19 lockdown compared to the pre-lockdown period. The survey included questions about time spent on sedentary activities, general physical activity, and specific play behaviors before and during the lockdown. It also assessed the parents' own physical activity and their involvement in their children’s activities. The relationships between these behaviors, as well as demographic and environmental factors, were analyzed.

Results

The study included 272 parents who completed an online survey about their child's physical activity during the COVID-19 lockdown. Many parents reported significant changes in their children's physical activity and screen time. Specifically, 89 (32.7%) observed a major decrease in physical activity, while 77 (28.3%) noted a minor decrease. Regarding screen time, 79 (29.0%) of parents reported a major increase, and 87 (32.0%) saw a minor increase. Key factors influencing children's physical activity were identified. Most notably, parents' own activity levels had a significant impact on their children's activity (p < 0.001). Additionally, children living in villas were more active than those living in apartments or traditional houses (p = 0.007), and UAE national children were slightly more active than non-nationals (p = 0.023).

Conclusion

This study demonstrated the significant impact of the COVID-19 lockdown on children’s activity and emphasized the importance of parental involvement in supporting their children's activity levels. It highlights the need for increased attention during the recovery phase from this crisis and calls for the development of strategies and guidelines to prevent similar challenges in future pandemics or similar emergencies.

## Introduction

The declaration of COVID-19 as a pandemic by the World Health Organization in early 2020 led to unprecedented global responses [1-5]. Countries worldwide implemented various preventative measures and isolation protocols to curb the spread of the virus [4,5]. Among the most drastic measures was the enforcement of total lockdowns, which significantly disrupted daily life. For children, this meant the loss of access to parks and school playgrounds, key venues for their daily physical activity. Instead, children were confined indoors, raising concerns about the emergence of a sedentary lifestyle and its potential health implications [6-8].

Studies have highlighted the adverse health consequences of a sedentary lifestyle, both immediately and in the long term. Short-term effects include reduced physical fitness, mood swings, and a negative impact on cognitive development, mental health, and social skills in young children [9,10]. Long-term effects include a higher risk of obesity, cardiovascular disease, and increased susceptibility to chronic health conditions later in life [11]. Conversely, an active lifestyle is associated with numerous health benefits, such as improved physical and emotional health, better stress management, and a stronger immune system [10,12-16]. Physical activity may also enhance school performance and learning ability [16], both of which are threatened by increased screen time and reduced physical activity during quarantine [17,18]. Additionally, increased screen use has been linked to several harmful effects on children's health, including sleep disturbances, lower self-esteem, an increased risk of depression and anxiety, and delayed development of language and social skills in younger children [8,18-20].

Several international studies provide valuable insights into this issue, highlighting the influence of various factors on children's physical activity [7,21-24]. For instance, a study conducted in Portugal explored the role of household characteristics, such as parental education and family income, in shaping children's activity levels [21]. These findings underscore the significance of socio-economic and familial contexts in promoting healthier lifestyles among children. In Canada, researchers compared children's activity levels before and during lockdown [7], while in the United States, shifts in daily routines, including screen time and sleep, were surveyed [22,23]. In Qatar, changes in diet were also explored [24]. A global review emphasized the role of parental influence on children's coping mechanisms during quarantine [25]. However, studies specific to the UAE remain limited. This research aims to assess the impact of the COVID-19 quarantine on children's physical activity levels in the UAE and analyze the factors influencing their activity during this period, providing new insights into the scale of the problem in the UAE.

## Materials and methods

Study design

This study employs a cross-sectional research design, as it is the most suitable and feasible for quantifying physical activity levels during the COVID-19 lockdown. A questionnaire was distributed among a specific population to achieve this objective (see Appendix A).

Population sample size

The study targets parents of children from several schools across different Emirates, including children 12 years of age or below, who have been in the UAE for at least a month during the lockdown, which began on the 8th of March 2020. Children with disabilities or medical conditions that limit their physical activity were excluded from the study. This study utilized a non-random convenience sampling method, as participants were recruited through online platforms where parents voluntarily responded to the survey link.

The sample size (n) was determined using the formula: $n=\frac{4P(1-P)}{ME^{2}}$.

The standard marginal error (ME) is 5%, and with no real evidence regarding the expected prevalence (P), we considered it 50% to maximize the required sample size and enhance accuracy. This calculation resulted in a sample size of 400. The sample was then increased to 450 to account for potential non-responses, which would only become evident during the data analysis phase.

Survey development and content

The questionnaire used in this study was adapted from a Canadian study that based its questions on health behaviors defined by the Canadian Movement Guidelines for Children [7]. Several adjustments were made to the questionnaire to better address the study's secondary objectives and suit the targeted UAE population. First, demographic questions were adjusted to include UAE-specific details, such as the nationality and emirate of residence, while excluding questions about postal codes, ethnicity, and dog ownership, which were not relevant to the study population. Second, the response format for certain questions was modified; for example, physical activity and screen time questions used categorical responses instead of open-ended numerical inputs to simplify data collection. Additionally, questions assessing changes in physical activity levels and family movement behaviors were refined by consolidating some categories. The final questionnaire consisted of 27 questions and was available in both Arabic and English to ensure accessibility. It was divided into three sections: (1) demographic characteristics of parents and children, (2) current movement behaviors over the past week, and (3) changes in physical activity levels compared to pre-quarantine. To encourage completion and minimize non-responses, the questionnaire primarily included Yes/No and multiple-choice questions, allowing for completion within 10 minutes.

Ethical consideration

Ethical clearance was obtained from the Office of Vice Chancellor for Research and Graduate Studies Research Ethics Committee, University of Sharjah (REC-21-02-12-03-S). Participation was voluntary, anonymous, and risk-free.

Data collection

Before the main data collection phase, a pilot study involving 10-15 participants was conducted in December 2020 to assess the clarity and feasibility of the questionnaire. Following this, the questionnaire, alongside a consent form in both Arabic and English, was distributed online during February, March, and April of 2021 through school WhatsApp groups involving parents of children of various educational levels and across different Emirates, in addition to other community pages and forums for parents of primary and middle-school children. Parents were requested to complete the survey once for each of their children under 12 years old. All collected data was anonymous, confidential, and used solely for research purposes.

Data analysis

Data analysis of the collected responses was conducted using SPSS 24 (IBM Corp., Released 2016. IBM SPSS Statistics for Windows, Version 24.0. Armonk, NY: IBM Corp.), employing univariate analysis to generate descriptive statistics, including frequency and central tendency measures. Bivariate analysis was also used to examine relationships between variables. The SPSS license was obtained through the University of Sharjah's institutional SPSS software license. The chi-square test was primarily used to evaluate association with a significance level of 5%.

## Results

Parent and child demographic characteristics

Out of a sample size of 272 children, more than half of the participants (60.7%) lived in villas, some lived in apartments (33.1%), and a small minority resided in traditional housing (6.3%). Most participants were Emirati (57.4%), some were non-Emirati Arabs (38.2%), and a few were non-Arabs (4.4%). Most children (54.8%) were in the age group 9-12, while the other age groups, under five and five to eight, constituted 16.9% and 28.3%, respectively. Regarding gender distribution, 52.9% were females, and 47.1% were males. Table 1 describes the demographic characteristics of the children involved in this study.

**Table 1:** Description of child characteristics (n = 272)

Category	n (%)
Child age	
<5 years	46 (16.9)
5-8 years	77 (28.3)
9-12 years	149 (54.8)
Child gender	
Female	144 (52.9)
Male	128 (47.1)
Emirate of residence	
Sharjah	115 (42.3)
Abu Dhabi	52 (19.1)
Ajman	39 (14.3)
Fujairah	29 (10.7)
Dubai	26 (9.6)
Ras Al Khaimah	8 (2.9)
Umm Al Quwain	3 (1.1)
Nationality	
Arab	104 (38.2)
Emirati	156 (57.4)
Non-Arab	12 (4.4)
Household type	
Villa	164 (60.7)
Apartment	90 (33.1)
Traditional housing	17 (6.3)

Changes in children’s physical activity compared to pre-COVID-19 habits

To evaluate children's physical activity levels during the COVID-19 lockdown, parents were asked to report how often their children engaged in physical activity each week. Most children (47.4%) were physically active for only one day per week. A smaller percentage (30.9%) engaged in physical activity for two to three days per week, and just 9.9% were active for three to five days per week. Only 11.8% of children participated in physical activity for more than five days a week, indicating a trend toward minimal physical activity during lockdown and a lack of regular exercise.

To further assess the shift toward sedentary behavior during this period, parents were also asked to report the number of hours their children engaged in activities such as watching TV, using the computer, using social media, and playing inactive video games each day. The majority of children (64%) spent five or more hours daily in sedentary activities, indicating a significant rise in inactivity. In contrast, only 11.4% of children spent three hours or less in sedentary behavior, suggesting they may have maintained higher activity levels than their counterparts. An additional 24.6% were inactive for three to four hours per day.

Additionally, the questionnaire assessed parents' physical activity to understand its potential influence on their children's behaviors during lockdown. The majority of parents reported low levels of activity; nearly half of them (47.4%) were only active for just one day per week, while 35.3% engaged in physical activity for two to three days, and 10.7% were active for three to five days. Only 6.6% of parents were physically active more than five days a week.

Changes in movement and play behaviors

Parents were also asked about the changes in their children's engagement with specific activities compared to the time before the COVID-19 outbreak. The questions covered various aspects of children's physical activities, including walking, sports, household chores, and play, alongside sedentary behaviors and other leisure activities. Parents rated changes in these habits on a scale from significantly less to significantly more.

For child physical activity and sports, 32.7% of parents reported a significant decrease, 28.3% reported a slight decrease, 24.6% reported no change, 8.8% reported a slight increase, and 5.5% reported a significant increase.

On the other hand, when rating child screen time, 4.8% of parents reported a significant decrease, 8.1% reported a slight decrease, 26.1% reported no change, 32.0% reported a slight increase, and 29.0% reported a significant increase.

**Figure 1:**
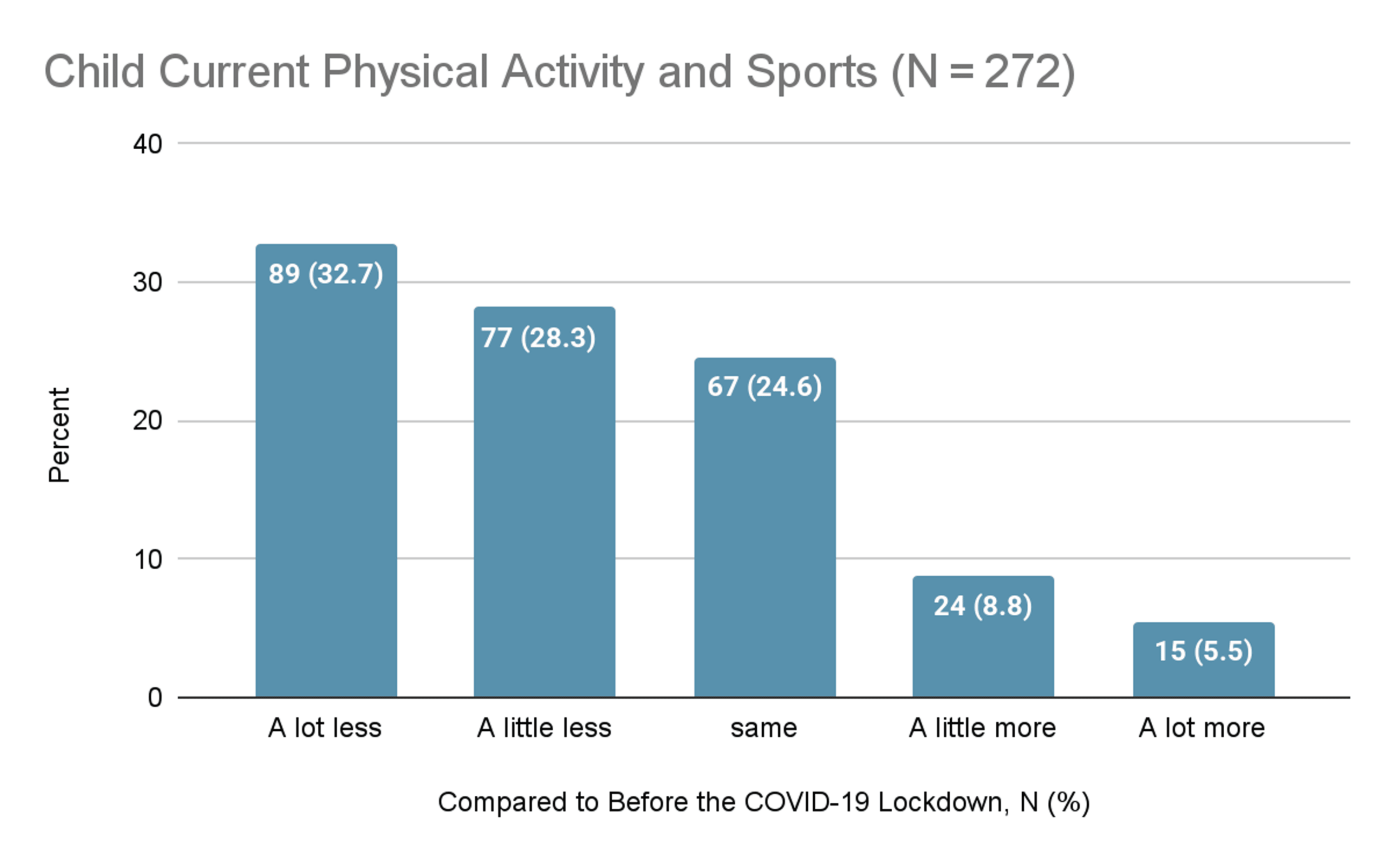
Changes in physical activity and sports

**Figure 2:**
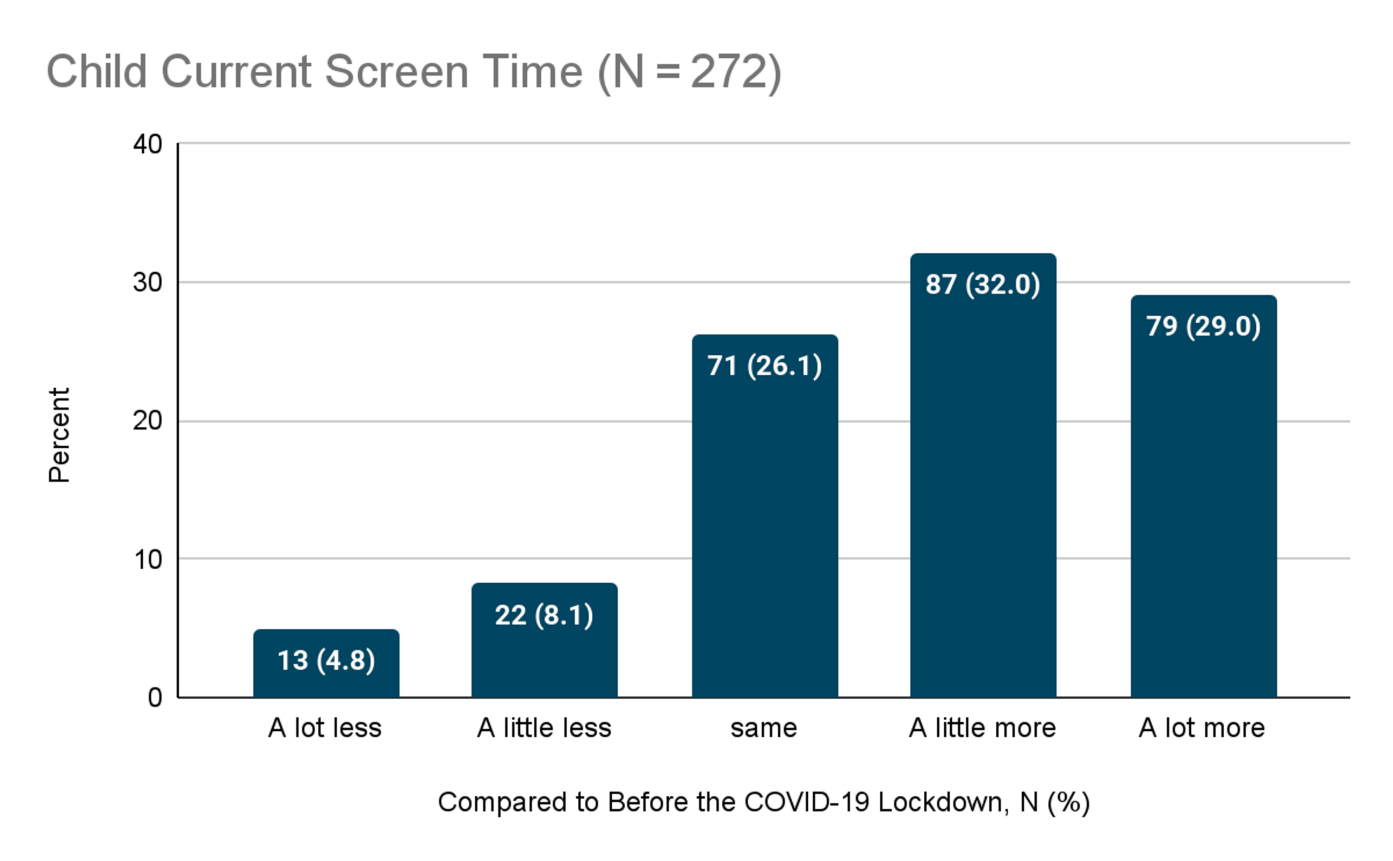
Changes in screen time

**Figure 3:**
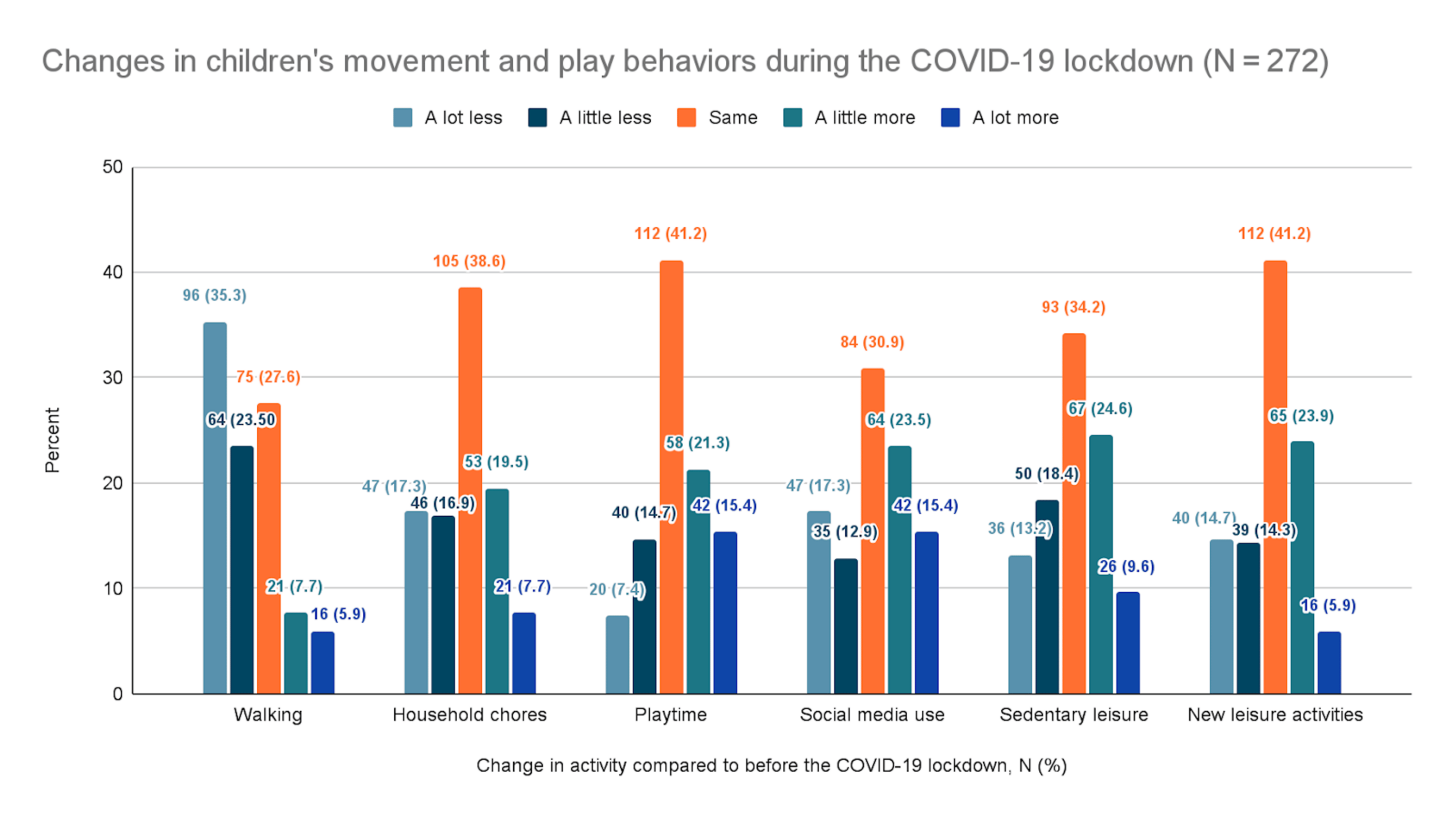
Changes in children's movement and play behaviors during the COVID-19 lockdown

Overall, there was a considerable decline in physical activity and sports and a marked increase in screen time. Other behaviors showed inconsistent patterns. Figure 1 depicts the patterns of change in physical activity and sports. Figure 2 depicts the patterns of change in screen time. Figure 3 summarizes the changes in other activities.

Changes in family behavior and habits

The questionnaire also assessed changes in family behavior toward physical activity and sedentary behavior, as well as the use of online resources and apps to support healthy movement.

Many families reported a decrease in physical activity following the COVID-19 quarantine, with 26.5% stating they were much less active and 27.6% noting they were slightly less active than before. In contrast, 18.0% reported a slight increase in family physical activity, while only 3.3% reported being significantly more active. A total of 24.6% of families stated that their physical activity levels remained the same as before the pandemic.

Furthermore, there was a notable increase in the time families spent practicing sedentary activities. A total of 19.1% of families reported a substantial increase, while 36.4% noted a slight increase in sedentary behavior. Meanwhile, only 8.1% of families reported a significant decrease in sedentary activity, and 12.9% reported a slight decrease. Based on the findings, 23.5% of families reported that their sedentary behavior remained unchanged compared to the period before the pandemic.

Parents were also asked about their families' engagement in new activities they had not tried before the COVID-19 lockdown. A large proportion of families (34.6%) reported no change in the frequency they engaged in novel activities compared to before COVID-19. A smaller group noticed slight changes, with 24.6% engaging in a little more novel activity and 21.0% doing a little less. Only a few families experienced major changes, with 15.4% reporting a notable decrease in practicing new activities and 4.4% reporting a significant increase.

A similar pattern was observed in families' use of online resources and apps to support healthy movement during the lockdown compared to before. Many families (34.9%) reported no change in usage, while 19.5% indicated they used these resources a little more and 18.0% a little less. Fewer families reported significant changes, with 20.2% using them a lot less and only 7.4% using online resources and apps more than before the pandemic to support movement and activity.

Decrease in child health

Most parents observed no noticeable decrease in their child's health after spending time in quarantine (88.6%). Among the parents who reported a worsening state of their child's health (11.4%), the most commonly reported morbidity was an increase in the child's weight (63%). This weight increase was based on self-reported observations from parents. Other reported health issues included tiredness (29.6%) and general sickness (7.4%).

Parents' views on the importance of physical activity

When asked about the importance of physical activity for their children, 90.4% of the participants considered it very important, 7.7% viewed it as important, and 1.8% rated it as slightly important. This suggests that most parents recognize the significant role of physical activity in supporting their children's health and overall well-being.

Bivariate analysis of factors influencing physical activity

Demographics in Relation to Physical Activity

Provided are tables with various demographic factors and their association with physical activity. The age of the child showed no association with their level of activity (p = 0.126), as shown in detail in Table 2. No significant association was seen between the gender of the child and their physical activity (p = 0.257), as seen in Table 3 in detail. There was a significant relationship between nationality and the level of physical activity in children, with local children participating in physical activity on more days than non-local children (p = 0.023), with details depicted in Table 4. A significant association was observed between the type of housing a child lived in and their level of physical activity, with children residing in villas showing higher levels of physical activity compared to those living in apartments and traditional houses (p = 0.007), as shown in Table 5. Despite differences in activity patterns across the Emirates, the bivariate analysis indicated that the Emirate of residence did not have a statistically significant impact on a child's physical activity (p = 0.510), as reported in Table 6.

**Table 2:** Bivariate analysis of child's physical activity level by age group Chi-square test results: χ² = 9.957^a^, p-value = 0.126 ^a^ One cell (8.3%) has an expected count of less than 5. The minimum expected count is 4.57.

			Children age groups			Total
			<5	5-8	9-12	
Number of days active per week (child)	1 day	Count	25	26	78	129
		Number of days active per week (%)	19.40	20.20	60.50	100.00
		% within age group	54.30	33.80	52.30	47.40
		% of total	9.20	9.60	28.70	47.40
	2-3 days	Count	10	32	42	84
		Number of days active per week (%)	11.90	38.10	50.00	100.00
		% within age group	21.70	41.60	28.20	30.90
		% of total	3.70	11.80	15.40	30.90
	3-5 days	Count	4	9	14	27
		Number of days active per week (%)	14.80	33.30	51.90	100.00
		% within age group	8.70	11.70	9.40	9.90
		% of total	1.50	3.30	5.10	9.90
	more than 5 days	Count	7	10	15	32
		Number of days active per week (%)	21.90	31.30	46.90	100.00
		% within age group	15.20	13.00	10.10	11.80
		% of total	2.60	3.70	5.50	11.80
Total		Count	46	77	149	272
		Number of days active per week (%)	16.90	28.30	54.80	100.00
		% within age group	100.00	100.00	100.00	100.00
		% of total	16.90	28.30	54.80	100.00

**Table 3:** Bivariate analysis of child's physical activity level by gender Chi-square test results: χ² = 4.037^a^, p-value = 0.257 ^a^ 0 cells (0.0%) have an expected count of less than 5. The minimum expected count is 12.71.

			Gender of child		Total
			Female	Male	
Number of days active per week (child)	1 day	Count	71	58	129
		Number of days active per week (child) (%)	55.00	45.00	100.00
		% within gender of child	49.30	45.30	47.40
		% of total	26.10	21.30	47.40
	2-3 days	Count	48	36	84
		Number of days active per week (child) (%)	57.10	42.90	100.00
		% within gender of child	33.30	28.10	30.90
		% of total	17.60	13.20	30.90
	3-5 days	Count	10	17	27
		Number of days active per week (child) (%)	37.00	63.00	100.00
		% within gender of child	6.90	13.30	9.90
		% of total	3.70	6.30	9.90
	more than 5 days	Count	15	17	32
		Number of days active per week (child) (%)	46.90	53.10	100.00
		% within gender of child	10.40	13.30	11.80
		% of total	5.50	6.30	11.80
Total		Count	144	128	272
		Number of days active per week (child) (%)	52.90	47.10	100.00
		% within gender of child	100.00	100.00	100.00
		% of total	52.90	47.10	100.00

**Table 4:** Bivariate analysis of child's physical activity level by nationality Chi-square test results: χ² = 9.561^a^, p-value = 0.023 ^a^ 0 cells (0.0%) have an expected count less of than 5. The minimum expected count is 11.51.

		Nationality grouped		Total
		Non-Local	Local	
1 day	Count	63	66	129
	Number of days active per week (child) (%)	48.80	51.20	100.00
	% within nationality grouped	54.30	42.30	47.40
	% of total	23.20	24.30	47.40
2-3 days	Count	35	49	84
	Number of days active per week (child) (%)	41.70	58.30	100.00
	% within nationality grouped	30.20	31.40	30.90
	% of total	12.90	18.00	30.90
3-5 days	Count	12	15	27
	Number of days active per week (child) (%)	44.40	55.60	100.00
	% within nationality grouped	10.30	9.60	9.90
	% of total	4.40	5.50	9.90
More than 5 days	Count	6	26	32
	Number of days active per week (child) (%)	18.80	81.30	100.00
	% within nationality grouped	5.20	16.70	11.80
	% of total	2.20	9.60	11.80
Total	Count	116	156	272
	Number of days active per week (child) (%)	42.60	57.40	100.00
	% within nationality grouped	100.00	100.00	100.00
	% of total	42.60	57.40	100.00

**Table 5:** Bivariate analysis of child's physical activity level by form of residence Chi-square test results: χ² = 17.791^a^, p-value = 0.007 ^a^ Two cells (16.7%) have an expected count of less than 5. The minimum expected count is 1.69.

			Form of residence			Total
			Villa	Apartment	Traditional	
Number of days active per week (child)	1 day	Count	64	53	12	129
		Number of days active per week (child) (%)	49.60	41.10	9.30	100.00
		% within form of residence	38.80	58.90	70.60	47.40
		% of total	23.50	19.50	4.40	47.40
	2-3 days	Count	54	27	3	84
		Number of days active per week (child) (%)	64.30	32.10	3.60	100.00
		% within form of residence	32.70	30.00	17.60	30.90
		% of total	19.90	9.90	1.10	30.90
	3-5 days	Count	20	6	1	27
		Number of days active per week (child) (%)	74.10	22.20	3.70	100.00
		% within form of residence	12.10	6.70	5.90	9.90
		% of total	7.40	2.20	0.40	9.90
	more than 5 days	Count	27	4	1	32
		Number of days active per week (child) (%)	84.40	12.50	3.10	100.00
		% within form of residence	16.40	4.40	5.90	11.80
		% of total	9.90	1.50	0.40	11.80
Total		Count	165	90	17	272
		Number of days active per week (child) (%)	60.70	33.10	6.30	100.00
		% within form of residence	100.00	100.00	100.00	100.00
		% of total	60.70	33.10	6.30	100.00

**Table 6:** Bivariate analysis of Emirate of residence and child's physical activity level Chi-square test results: χ² = 17.191^a^, p-value = 0.51 ^a^ 14 cells (50.0%) have an expected count of less than 5. The minimum expected count is 0.30.

			Emirate of Residence							Total
			Ajman	Sharjah	Abu Dhabi	Dubai	Fujairah	Ras Al Khaimah	Umm Al Quwain	
Number of days active per week (child)	1 day	Count	16	64	16	13	14	4	2	129
		Number of days active per week (child) (%)	12.40	49.60	12.40	10.10	10.90	3.10	1.60	100.00
		% within Emirate	41.00	55.70	30.80	50.00	48.30	50.00	66.70	47.40
		% of total	5.90	23.50	5.90	4.80	5.10	1.50	0.70	47.40
	2-3 days	Count	14	27	21	9	10	2	1	84
		Number of days active per week (child) (%)	16.70	32.10	25.00	10.70	11.90	2.40	1.20	100.00
		% within Emirate	35.90	23.50	40.40	34.60	34.50	25.00	33.30	30.90
		% of total	5.10	9.90	7.70	3.30	3.70	0.70	0.40	30.90
	3-5 days	Count	4	10	7	3	1	2	0	27
		Number of days active per week (child) (%)	14.80	37.00	25.90	11.10	3.70	7.40	0.00	100.00
		% within Emirate	10.30	8.70	13.50	11.50	3.40	25.00	0.00	9.90
		% of total	1.50	3.70	2.60	1.10	0.40	0.70	0.00	9.90
	more than 5 days	Count	5	14	8	1	4	0	0	32
		Number of days active per week (child) (%)	15.60	43.80	25.00	3.10	12.50	0.00	0.00	100.00
		% within Emirate	12.80	12.20	15.40	3.80	13.80	0.00	0.00	11.80
		% of total	1.80	5.10	2.90	0.40	1.50	0.00	0.00	11.80
Total		Count	39	115	52	26	29	8	3	272
		Number of days active per week (child) (%)	14.30	42.30	19.10	9.60	10.70	2.90	1.10	100.00
		% within Emirate	100.00	100.00	100.00	100.00	100.00	100.00	100.00	100.00
		% of total	14.30	42.30	19.10	9.60	10.70	2.90	1.10	100.00

Physical Activity of Parents and Its Impact on Children’s Physical Activity

The activity of parents had a profoundly positive impact on the activity of their children, demonstrating a major association (p < 0.001), with highly active parents being much more likely to have similarly active children, while minimally active parents tended to have less active children. Described in detail in Table 7.

**Table 7:** Bivariate analysis of child's physical activity level by parental physical activity Chi-square test results: χ² = 52.872^a^, p-value = 0.000 ^a^ One cell (8.3%) has an expected count of less than 5. The minimum expected count is 4.67.

			Number of days active (parent)			Total
			1 day	2-3 days	more than 3 days	
Number of days active per week (child)	1 day	Count	84	34	11	129
		Number of days active per week (child) (%)	65.10	26.40	8.50	100.00
		Number of days active per week (parent) (%)	65.10	35.40	23.40	47.40
		% of total	30.90	12.50	4.00	47.40
	2-3 days	Count	32	40	12	84
		Number of days active per week (child) (%)	38.10	47.60	14.30	100.00
		Number of days active per week (parent) (%)	24.80	41.70	25.50	30.90
		% of total	11.80	14.70	4.40	30.90
	3-5 days	Count	5	13	9	27
		Number of days active per week (child) (%)	18.50	48.10	33.30	100.00
		Number of days active per week (parent) (%)	3.90	13.50	19.10	9.90
		% of total	1.80	4.80	3.30	9.90
	more than 5 days	Count	8	9	15	32
		Number of days active per week (child) (%)	25.00	28.10	46.90	100.00
		Number of days active per week (parent) (%)	6.20	9.40	31.90	11.80
		% of total	2.90	3.30	5.50	11.80
Total		Count	129	96	47	272
		Number of days active per week (child) (%)	47.40	35.30	17.30	100.00
		Number of days active per week (parent) (%)	100.00	100.00	100.00	100.00
		% of total	47.40	35.30	17.30	100.00

Children’s Physical Activity and Weight Changes During Lockdown

The findings in Table 8 suggest that there is a statistically significant relationship between a child's weight and their level of physical activity (p = 0.025). Children who maintained stable weight during lockdown were generally more active, while those who gained weight were more likely to have lower levels of physical activity.

**Table 8:** Bivariate analysis of active days per week and weight changes relative to pre-COVID-19 weight Chi-square test results: χ² = 14.465^a^, p-value = 0.025 ^a^ One cell (8.3%) has an expected count of less than 5. The minimum expected count is 4.57.

			Child weight compared to pre-COVID-19 weight			Total
			Less	Same	More	
Number of days active per week (child)	1 day	Count	21	52	56	129
		Number of days active per week (child) (%)	16.30	40.30	43.40	100.00
		% within compared to before COVID-19 child weight	45.70	44.40	51.40	47.40
		% of total	7.70	19.10	20.60	47.40
	2-3 days	Count	13	31	40	84
		Number of days active per week (child) (%)	15.50	36.90	47.60	100.00
		% within compared to before COVID-19 child weight	28.30	26.50	36.70	30.90
		% of total	4.80	11.40	14.70	30.90
	3-5 days	Count	6	12	9	27
		Number of days active per week (child) (%)	22.20	44.40	33.30	100.00
		% within compared to before COVID-19 child weight	13.00	10.30	8.30	9.90
		% of total	2.20	4.40	3.30	9.90
	more than 5 days	Count	6	22	4	32
		Number of days active per week (child) (%)	18.80	68.80	12.50	100.00
		% within compared to before COVID-19 child weight	13.00	18.80	3.70	11.80
		% of total	2.20	8.10	1.50	11.80
Total		Count	46	117	109	272
		Number of days active per week (child) (%)	16.90	43.00	40.10	100.00
		% within compared to before COVID-19 child weight	100.00	100.00	100.00	100.00
		% of total	16.90	43.00	40.10	100.00

## Discussion

The results of this study unequivocally show that children's levels of physical activity significantly decreased during the COVID-19 lockdown, while sedentary behaviors, particularly screen time, significantly increased. In particular, 32.7% of parents reported that their children's physical activity had decreased considerably, while another 28.3% thought it had slightly decreased. This pattern is consistent with similar accounts around the world during the epidemic [21-24], indicating that prolonged indoor confinement significantly reduced children's possibilities for physical play and exercise. Simultaneously, a significant spike in screen usage was noted, with 32.0% of parents reporting a modest increase and 29.0% suggesting a major increase. Given that kids were restricted to their homes, this change probably reflects the growing reliance on digital gadgets for both education and amusement. Given its well-established links to detrimental health consequences in children, such as decreased physical fitness, poor sleep quality, and an increased risk of obesity [8,17-20], the rise in screen time is especially worrisome.

It is important to mention that while our findings suggest a significant effect of screen time on physical activity, other studies have shown that screen time involving quality content can have beneficial effects on children and that the focus should be less on screen time and more on the content being consumed. Screen time was shown to provide unique and enhanced opportunities for learning and education, especially with limited access to outside means, it also was the primary method of school teaching in countries that closed down schools during the lockdown with online physical education classes providing a positive influence on children’s engagement in physical activity during the pandemic [22,26]. The availability of internet use also played a crucial role in maintaining social connections and emotional support for children and adolescents in an era where offline socialization opportunities are limited [26,27].

The study identified key factors affecting children's levels of physical activity during lockdown, providing valuable insight into potential intervention strategies. The most significant factor (p < 0.001) was parental activity, as children of active parents were more likely to maintain higher levels of activity during quarantine. These findings highlight the importance of parental involvement in the physical activity of children, as parents may serve as role models and help mitigate some of the negative impacts of limited outdoor access. This is supported by the general literature showing a similarly strong association [7,24,28]. Consistent with past studies [7,21,29], the type of housing was another important predictor; children living in villas were more physically active than those living in apartments (p = 0.007). This might be explained by the greater availability of private outdoor areas in villa settings, allowing children to play outside without violating lockdown restrictions. Lastly, a small but statistically significant difference in activity levels by country was observed, with children from the local area being more active than those from other countries (p = 0.023). In addition to potentially varying degrees of access to communal or residential physical activity facilities, this disparity may result from cultural variables or varying family attitudes toward physical activity.

The validity of this study's conclusions is increased by several key strengths. A thorough evaluation of several aspects of children's physical behaviors, including screen time, specific play behaviors, and overall levels of physical activity, as well as parental engagement, was made possible by the use of a comprehensive survey. The study offers a thorough understanding of the social and environmental factors influencing children's physical activity during a public health emergency by incorporating data on demographic characteristics and types of housing. Furthermore, studying a diverse sample from multiple Emirates improves the generalizability of the findings and provides insights that might be relevant in other residential and cultural circumstances.

However, it is important to recognize certain limitations. Recall bias (where parents may misremember their children's pre-lockdown activity levels) and social desirability bias (where parents may overreport positive behaviors such as participation in physical activities) are potential biases inherent in self-reported data, limiting the objectivity in determining physical activity measures. Additionally, the cross-sectional design of the study also makes it difficult to establish causality. While strong associations were found between parental activity and children's physical activity levels, longitudinal studies are needed to establish causation. Moreover, the online nature of the survey, implemented due to COVID-19 restrictions, made it susceptible to sampling bias, as it likely reached parents with internet access and active social media engagement. This may have resulted in an over-representation of higher-income, digitally engaged families and an under-representation of families with lower socioeconomic status, limited internet access, or less involvement in online community groups. Furthermore, since participation was voluntary, this introduced self-selection bias, as parents who were more concerned or interested in their children's activity levels during the pandemic may have been more likely to respond, which could have resulted in an over-representation of certain viewpoints regarding children's physical activity levels during the pandemic. Finally, the survey did not account for additional factors that could have influenced children’s physical activity levels. For instance, only children tend to have more sedentary time compared to those with siblings [21]. Children living in higher socio-economic status areas generally demonstrate greater fitness levels and higher physical activity compared to those from lower socio-economic status areas [21]. Owning a pet dog has been associated with increased physical activity and outdoor time due to dog-related activities [7]. In addition, parental age may also play a role, with younger parents being linked to higher activity levels [7]. Furthermore, the disruptions to daily routines and increased stressors brought on by the COVID-19 pandemic may have negatively impacted children’s physical activity levels, as changes in sleep patterns and circadian timing are associated with lower physical activity [23].

## Conclusions

In conclusion, this study outlines the changes in children's activity and sedentary behaviors during the COVID-19 quarantine and emphasizes the vital role of family and environmental factors in mitigating the negative impacts of the lockdown measures on physical activity. These findings suggest that encouraging active parental participation and expanding access to secure play spaces, especially during periods of restricted mobility, may be useful tactics for supporting and maintaining children's physical activity. To translate these insights into practical action, public health policies could implement school-based interventions that encourage joint physical activities for parents and children, as well as urban planning initiatives that increase the availability of safe, accessible play areas. Additionally, community-driven campaigns encouraging family-centered physical activities and the development of flexible indoor exercise resources could help preserve activity levels during future periods of restricted mobility.

## Appendices

Appendix A

The questionnaire used in this study was adapted from a Canadian study with similar objectives [7], which was originally based on the health behaviors outlined in the Canadian Movement Guidelines for Children. It was modified to address the secondary objectives of this study and better reflect the population of the United Arab Emirates. Below are the survey questions

Demographic characteristics

Parents:

1- Which emirate do you live in? / في أي إمارة تقيم؟

A: Abu Dhabi, Dubai, Ajman, Fujairah, Ras Al Khaimah, Sharjah, Umm Al Quwain / أبوظبي، دبي، عجمان، الفجيرة، رأس الخيمة، الشارقة، أم القيوين

2- Types of residence / ما نوع السكن الذي تقطنه؟

A: Villa, Apartment / فيلا, شقة

3- What is your nationality? / ما هي جنسيتك؟

A: ………

4- Number of children / كم طفل لديك

A: 1-6

Child:

5- Age of child / كم عمر طفلك

A: ………

6- Gender of child / هل الطفل ذكر أم أنثى؟

A: Male, Female

7- Disability/Chronic condition / هل يعاني طفلك من أي حالة مرضية أو إعاقة جسدية؟

A: Yes / No

8- If yes, specify: ………

Current movement behaviors

9- In the last week, how many total hours and minutes per day did your child watch TV, use the computer, use social media, and play inactive video games? / كم ساعة أمضى طفلك في استخدام الحاسوب أو مواقع التواصل الاجتماعي أو الألعاب الإلكترونية ومشاهدة التلفاز خلال الأسبوع الفائت؟

A: Less than an hour, 1-2, 3-4, 5 or more

10- In the last week, on how many days did your child engage in moderate-to-vigorous physical activity for a total of at least an hour per day? / كم يوما مارس طفلك رياضة معتدلة أو قاسية لمدة ساعة في الأسبوع الفائت؟

A: 1 day, 2-3 days, 4-5 days, 6 days or more

11- In the last week, on how many days did you engage in moderate-to-vigorous physical activity for a total of at least an hour per day? / كم يوم مارست رياضة معتدلة أو قاسية لمدة ساعة في الأسبوع الماضي؟

A: 1 day, 2-3 days, 4-5 days, 6 days or more

Changes in movement and play behaviors compared to before the COVID-19 outbreak and related restrictions (child)

12- My child walks or bikes in the neighborhood / يمارس طفلي المشي أو ركوب الدراجة في الحي

A: A Lot Less (أقل بكثير), A Little Less (أقل بقليل), Same (كالمعتاد), A Little More (أكثر بقليل), A Lot More (أكثر بكثير)

13- My child is doing physical activities or sport / يقوم طفلي بممارسة النشاط البدني

A: A Lot Less (أقل بكثير), A Little Less (أقل بقليل), Same (كالمعتاد), A Little More (أكثر بقليل), A Lot More (أكثر بكثير)

14- My child is doing household chores (cleaning, yard work) / يشارك طفلي في الأعمال المنزلية

A: A Lot Less (أقل بكثير), A Little Less (أقل بقليل), Same (كالمعتاد), A Little More (أكثر بقليل), A Lot More (أكثر بكثير)

15- My child plays indoors / يلعب طفلي في المنزل

A: A Lot Less (أقل بكثير), A Little Less (أقل بقليل), Same (كالمعتاد), A Little More (أكثر بقليل), A Lot More (أكثر بكثير)

16- My child watches TV, movies, uses the computer for leisure / يمارس طفلي مشاهدة التلفاز واستخدام الحاسوب في وقت الفراغ

A: A Lot Less (أقل بكثير), A Little Less (أقل بقليل), Same (كالمعتاد), A Little More (أكثر بقليل), A Lot More (أكثر بكثير)

17- My child uses social media / يستخدم طفلي مواقع التواصل الاجتماعي

A: A Lot Less (أقل بكثير), A Little Less (أقل بقليل), Same (كالمعتاد), A Little More (أكثر بقليل), A Lot More (أكثر بكثير)

18- My child does other sedentary leisure activities not in front of screens / يمارس طفلي الأنشطة وهو جالس

A: A Lot Less (أقل بكثير), A Little Less (أقل بقليل), Same (كالمعتاد), A Little More (أكثر بقليل), A Lot More (أكثر بكثير)

19- Is there a leisure activity or hobby that your child is doing a lot more? / هل زادت ممارسة طفلك لهواية معينة؟

A: A Lot Less (أقل بكثير), A Little Less (أقل بقليل), Same (كالمعتاد), A Little More (أكثر بقليل), A Lot More (أكثر بكثير)

20- Did you notice any increase/decrease in your child’s weight? / هل لاحظت تغيرا في وزن طفلك؟

A: A Lot Less (أقل بكثير), A Little Less (أقل بقليل), Same (كالمعتاد), A Little More (أكثر بقليل), A Lot More (أكثر بكثير)

Changes in movement and play behaviors compared to before the COVID-19 outbreak and related restrictions (family)

21- Our family time spent in physical activity is / الوقت الذي تقضيه عائلتي بممارسة اللياقة البدنية

A: A Lot Less (أقل بكثير), A Little Less (أقل بقليل), Same (كالمعتاد), A Little More (أكثر بقليل), A Lot More (أكثر بكثير)

22- Our family time spent in sedentary behavior is / الوقت الذي تقضيه عائلتي في الجلوس

A: A Lot Less (أقل بكثير), A Little Less (أقل بقليل), Same (كالمعتاد), A Little More (أكثر بقليل), A Lot More (أكثر بكثير)

23- Has your family begun any new or novel activities not previously practiced? / هل تقوم عائلتك بممارسة أي نشاطات جديدة لم تقوموا بممارستها من قبل؟

A: A Lot Less (أقل بكثير), A Little Less (أقل بقليل), Same (كالمعتاد), A Little More (أكثر بقليل), A Lot More (أكثر بكثير)

24- Has your family used any online resources/apps to support healthy movement? هل تستعينون بأي مواقع الكترونية أو تطبيقات تساعدكم على ممارسة /الرياضة بشكل أفضل؟

A: A Lot Less (أقل بكثير), A Little Less (أقل بقليل), Same (كالمعتاد), A Little More (أكثر بقليل), A Lot More (أكثر بكثير)

25- Has there been a decrease in your child’s health (existing condition worsened/new condition developed)? / هل كان هناك انخفاض في صحة طفلك (ساءت الحالة الحالية / تطورت حالة جديدة)؟

A: Yes: (If yes, explain why) / No

26- How important do you think physical activity is important for your children? / برأيك، ما أهمية النشاط البدني لصحة أطفالك؟

A: Very important, important, slightly important, not important
